# Assessing post-abortion care using the WHO quality of care framework for maternal and newborn health: a cross-sectional study in two African hospitals in humanitarian settings

**DOI:** 10.1186/s12978-024-01835-9

**Published:** 2024-08-05

**Authors:** Estelle Pasquier, Onikepe O. Owolabi, Bill Powell, Tamara Fetters, Richard Norbert Ngbale, Daphne Lagrou, Claire Fotheringham, Catrin Schulte-Hillen, Huiwu Chen, Timothy Williams, Ann M. Moore, Mariette Claudia Adame Gbanzi, Pierre Debeaudrap, Veronique Filippi, Lenka Benova, Olivier Degomme

**Affiliations:** 1 Epicentre – Médecins Sans Frontières, Paris, France; 2 Department of Public Health and Primary Care, Ghent University, Ghent, Belgium; 3 Department of Public Health - Institute of Tropical Medicine, Antwerp, Belgium; 4 Guttmacher Institute, New York, NY USA; 5 Ipas, Chapel Hill, NC USA; 6 Ministère de la Santé et de la Population de la République Centrafricaine, Bangui, Central African Republic; 7 Médecins Sans Frontières, Brussels, Belgium; 8 Médecins Sans Frontières, Sydney, Australia; 9 Médecins Sans Frontières International, Geneva, Switzerland; 10 Epicentre - Médecins Sans Frontières, Abuja, Jigawa State Nigeria; 11 CEPED, Institut de Recherche pour le Développement, Université Paris Descartes, INSERM 1244, Paris, France; 12 Faculty of Epidemiology and Population Health - London School of Hygiene and Tropical Medicine, London, UK

**Keywords:** Maternal health, Quality of care, Abortion, Postabortion care, Hospital, Humanitarian, Nigeria, Central African Republic

## Abstract

**Background:**

Abortion-related complications remain a main cause of maternal mortality. There is little evidence on the availability and quality of post-abortion care (PAC) in humanitarian settings. We assessed the quality of PAC in two hospitals supported by an international organization in Jigawa State (Nigeria) and Bangui (Central African Republic, CAR).

**Methods:**

We mapped indicators corresponding to the eleven domains of the WHO Maternal and Newborn Health quality-of-care framework to assess inputs, processes (provision and experience of care), and outcomes of PAC. We measured these indicators in four components of a cross-sectional multi-methods study: 1) an assessment of the hospitals’ PAC signal functions, 2) a survey of the knowledge, attitudes, practices, and behavior of 140 Nigerian and 84 CAR clinicians providing PAC, 3) a prospective review of the medical records of 520 and 548 women presenting for abortion complications and, 4) a survey of 360 and 362 of these women who were hospitalized in the Nigerian and CAR hospitals, respectively.

**Results:**

Among the total 27 PAC signal functions assessed, 25 were available in the Nigerian hospital and 26 in the CAR hospital. In both hospitals, less than 2.5% were treated with dilatation and sharp curettage. Over 80% of women received blood transfusion or curative antibiotics when indicated. However, antibiotics were given to about 30% of patients with no documented indication. Among discharged women in CAR, 99% received contraceptive counseling but only 39% did in Nigeria. Over 80% of women in Nigeria reported positive experiences of respect and preservation of dignity. Conversely, in CAR, 37% reported that their privacy was always respected during examination and 62% reported short or very short waiting time before seeing a health provider. In terms of communication, only 15% felt able to ask questions during treatment in both hospitals. The risk of abortion-near-miss happening ≥ 24h after presentation was 0.2% in Nigeria and 1.1% in CAR. Only 65% of women in the Nigerian hospital and 34% in the CAR hospital reported that the staff provided them best care all the time.

**Conclusion:**

Our comprehensive assessment identified that these two hospitals in humanitarian settings provided lifesaving PAC. However, hospitals need to strengthen the patient-centered approach engaging patients in their own care and ensuring privacy, short waiting times and quality provider-patient communication. Health professionals would benefit from instituting antibiotic stewardships to prevent antibiotic-resistance.

**Supplementary Information:**

The online version contains supplementary material available at 10.1186/s12978-024-01835-9.

## Abstract (plain language summary)

In humanitarian contexts, abortion complications are a leading cause of maternal mortality. Providing quality post-abortion care (PAC) is therefore an important part of needed services. We assessed the quality of PAC in two hospitals supported by an international organization in Jigawa State (Nigeria) and Bangui (Central African Republic). We measured quality indicators in four components: 1) an assessment of the equipment and human resources available in hospitals, 2) a survey of the knowledge, attitudes, practices, and behavior of clinicians providing PAC, 3) an assessment of the medical care provided by clinicians to women presenting with abortion complications and, 4) a survey of a subgroup of these women who were hospitalized. Both hospitals had almost all the equipment and human resources necessary to provide post-abortion care. Less than 2.5% of women received a non-recommended method to evacuate their uterus in both hospitals. More than 80% of women received a blood transfusion or antibiotics when they needed them. However, 30% of women received antibiotics without written justification and only 15% of women reported being able to ask questions about their treatment. Overall, only 65% of Nigerian women and 34% of Central African women said that the staff provided them with the best care all the time. The fact that less than 2% of women experienced a very severe complication 24 hours or more after their arrival at the two hospitals suggests that the care provided was lifesaving. But they urgently need to adopt a better patient-centered approach as well as to improve the rational management of antibiotics.

**Supplementary Information:**

The online version contains supplementary material available at 10.1186/s12978-024-01835-9.

## Background

Abortion-related complications account for between 8 and 18% of global maternal deaths [1, 2], and result mainly from unsafe abortions [3]. Although most abortion-related deaths are preventable through access to contraception, safe abortion and post-abortion care, abortion-related mortality showed one of the smallest declines among all direct causes of maternal death between 1990 and 2017 [4]. Even in countries where access to safe abortion is restricted by law, emergency care for women with abortion complications (post-abortion care, PAC) is instrumental to save lives and prevent morbidity. In humanitarian settings, the need for high quality PAC is likely greater than in stable settings. In such contexts, the maternal mortality ratio is estimated to be twice the global average [5], with abortion-related complications among the five main causes [5]. The disruption of health systems affecting availability of and access to routine contraceptive and safe abortion services [6], as well as the higher risk of exposure to sexual violence and transactional sex can increase the risk of unwanted pregnancies leading to unsafe abortion [7]. Therefore, ensuring provision of high quality PAC is critical in these contexts [8].

Quality of care is a multi-dimensional concept [9, 10] making it difficult to measure comprehensively. The Donabedian model [9] is frequently used to operationalize the definition of quality because it is widely accepted as encompassing the main components of health care quality [11]. It includes three dimensions: structure or inputs to care; process of care; and (health) outcomes of care. Several quality-of-care frameworks incorporating the three Donabedian dimensions have been developed to drive quality improvement processes [12], including for maternal care [10, 13–15] and safe abortion care [16, 17]. The World Health Organization (WHO) conceptual framework for maternal and newborn health care (MNHC) extends the Donabedian’s model by dividing the process of care into the provision of care by health professionals and women’s experience of care to emphasize the importance of people-centered care [10].

Few studies have evaluated the quality of PAC in humanitarian settings [18–25]. Those that have been published have typically assessed only a specific aspect of quality of care: either care inputs (facility equipment, supplies and human resources capacity to provide PAC) [18, 20, 22], provision of care (number of PAC patients, manual vacuum aspiration (MVA) use and contraception uptake) [24], patients’ experience of care [21] or a combination of the latter two components of the process of care [23, 25]. Only one study assessed indicators of the three Donabedian dimensions [19]. Given the significant burden of abortion complications in humanitarian settings [26], comprehensive measurement of quality of PAC is essential to enable providers, facilities, and programmatic staff to assess and improve the quality-of-care women receive and ensure the best possible health outcomes after treatment. Thus, the objective of this study was to assess comprehensively the three Donabedian quality dimensions of PAC provided in two hospitals of humanitarian settings: one in Bangui in the Central African Republic (CAR) and one in Jigawa State in northern Nigeria.

## Methods

### Study design and population

This study was part of the AMoCo (**A**bortion-related **Mo**rbidity and mortality in fragile and **Co**nflict-affected settings) study. We measured the quality of PAC in four components of this cross-sectional multi-methods study with a prospective data collection: 1) a health facility assessment, 2) a knowledge, attitudes, practices, and behavior (KAPB) survey of health professionals, 3) a prospective medical records review of women presenting with abortion-related complications in the two hospitals, and 4) a quantitative patient survey among a sample of hospitalized women. The AMoCo study protocol, including the study design, each component detailed procedures, sampling, informed consent processes and other ethical considerations is available in the Médecins Sans Frontières (MSF science portal [27] and is registered with ClinicalTrials.gov NCT04331847.

### Study sites

The study was conducted in two MSF-supported referral hospitals in humanitarian settings described elsewhere [26] and in Additional file 1. Briefly, the hospital in the Central African Republic is situated in Bangui, the country’s capital, in an area affected by decades of armed conflicts [28]. During the pregnancies of women included in the study (April 2019 to January 2020), Bangui recorded 5.7 conflict-related deaths/year/100 000 persons [29] classifying them as medium-intensity conflict-affected settings by the World Bank [30]. The hospital in Northern Nigeria is located in Jigawa State, a fragile rural State that reported frequent intense floodings (14 episodes that last an average of 64 h/month [31]), a Lassa fever outbreak [32], and kidnappings and influx of displaced population because of armed conflicts in neighboring States [33] during the pregnancies of women included in the study (August 2019 to July 2021). Each facility operated under the same MSF medical management guidelines, had around 10,000 deliveries per year, a catchment area of more than 500,000 people, and the capability to provide comprehensive emergency obstetric care. In the CAR hospital, contraceptive services were provided close to PAC services and under the same management team. PAC procedures systematically included a pre-discharged contraceptive counseling consultation. In the Nigerian hospital, contraceptive services were not integrated with PAC. They were provided in another department of the hospital, under a different management team.

### Selection of quality measures and indicators for PAC quality

**Fig. 1:**
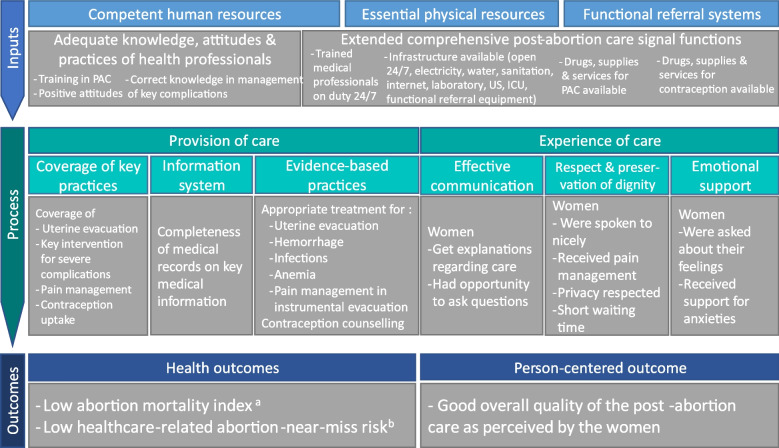
Framework for assessing the quality of PAC in hospitals, including 11 domains and 29 quality measures PAC: Post-Abortion Care, US: Ultra-Sound, ICU: Intensive Care Unit ^a^ Abortion-related mortality index = number of abortion-related deaths/ number of abortion-related near-miss cases and deaths ^b^ Risk of healthcare-related abortion-near-miss = number of women with abortion-related near-miss happening > = 24 h after presentation/total number of women presenting for abortion complications. Near-miss cases include women with organ dysfunction of either one or more of the following: cardiovascular, respiratory, renal, coagulation, hepatic, neurological or uterine dysfunction according to WHO criteria [38]

We used the WHO framework for MNHC with some modifications to include PAC specific indicators which encompass the three Donabedian dimensions. During the design of the AMoCo study, the technical steering group collaboratively modified some elements of the WHO quality of care framework for MNHC [10] to measure PAC quality in the two hospitals through a consensus meetings process [34] that is detailed in the technical note publicly available [35]. Briefly, the WHO framework includes eleven domains: competent human resources, essential physical resources, functional referral systems, coverage of key medical practices, actionable information system, evidence-based practices, effective communication, respect and preservation of dignity, emotional support as well as health and person-centered outcomes. For each domain, we identified key indicators of PAC quality based on literature reviews [35] and WHO and MSF PAC guidelines [36, 37]. Indicators were chosen by consensus between the technical steering members with clinical backgrounds, clinical experience in hospitals of humanitarian settings and/or experience in measuring quality of care (OO, BP, CF, DL, TF, CSH, EP). We then classified them into the three dimensions of the Donabedian framework [9]. Figure 1 presents this quality framework with the 11 domains and a total of 29 PAC quality measures which are captured by the indicators described in Additional file 2.

Inputs, also called “structure”, were measured using an adapted and extended version of Campbell et al. PAC signal functions [39, 40] assessing the structural capability and readiness of a health facility to provide PAC. Signal functions are essential physical and human resources needed in a health facility to support key lifesaving interventions including adequate drugs, supplies, equipment, infrastructure and trained staff to competently provide the service [41]. Human resources competences were measured using questions about their KAPB built from MSF PAC guidelines [37]. We included two components of process of care: provision and experience of care. Provision of care indicators were proposed to measure 1) coverage of key medical practices, meaning the percentage of women who received these key practices, 2) completeness of key medical information in the women’s medical records as an indicator of the information system and 3) percentages of women who received key medical interventions according to MSF PAC guidelines [37] as indicators of evidence-based practices. The women’s experience of care related to effective communication, respect, preservation of dignity and emotional support was measured using questions from the validated person-centered maternity care scale [42–44] and from the questionnaire of the WHO Multi-Countries Study on Abortion (WHO-MCS-A) [45]. The percentage of women reporting that the staff took the best care of them all the time was chosen as the indicator of person-centered outcomes. Health outcomes included the facility-based abortion-related mortality index as defined by WHO [38] and the percentage of women having “healthcare-related near-miss” as proposed by FEMHealth [46]. The definitions of these two indicators are in Fig. 1.

### Procedures

During the design of the AMoCo study, we identified the variables needed to measure all indicators of the quality framework. Corresponding questions or items were included in four standardized data collection tools of the four components described above.

#### Health facility assessment

After informed consent, the heads of the gynecology/obstetrics ward of the two hospitals completed the facility assessment form on signal functions.

#### KAPB survey

All physicians, midwives, nurses, and midwifery/nursing assistants providing PAC in study hospitals and literate in English or French were invited and consented to complete a self-administered questionnaire. The survey took place prior to any introduction to the study with hospital clinicians and prior to the extraction of information from medical records to prevent response biases linked to clinicians’ involvement in the rest of the study.

#### Prospective medical record review

All women presenting to study hospitals with signs and/or symptoms of complications related to spontaneous or induced abortions were included. Women with threatened abortions were excluded. Eligible women were identified by the study clinicians through hospital registers tracking and participations to daily gynecologic-obstetrics clinical meetings [26]. After checking their eligibility, women were included in the study after an informed opt-out consent process. Study clinicians, specifically hired for the study and independent from hospitals’ clinicians, reviewed women’s medical records prospectively daily with the help of the hospitals’ clinician in charge of the woman’s care when information was missing. Data were extracted in the standardized Case Report Form adapted from the WHO-MCS-A [45]. It included socio-demographics and obstetric characteristics, presentation clinical characteristics, severity criteria including WHO near-miss criteria [38], the detailed medical management received, and the outcome.

#### Quantitative patient survey

The prospective medical records’ review was followed by an interview survey among women who stayed at least overnight. Before discharge, eligible women were identified by study clinicians and invited to participate by trained female interviewers when they were medically stabilized. These interviewers, specifically hired for the study and independent from the medical staff, took informed consent and conducted semi-directed face-to-face interviews in a private room. Questions in the survey included sociodemographic background, reproductive characteristics, and experience of care. The questionnaire was found to be too long after its pre-testing. As a result, questions of the emotional domain were chosen to be removed because they were identified as ambiguous in one of the validation studies of the Person-Centered Maternity Scale [43]. Our questionnaire was designed in English and was translated into French and local languages and then back translated into English.

Data were collected between August 2019 and July 2021 in the Nigerian hospital (with an interruption between April and July 2020 due to COVID-19) and between September 2019 and January 2020 in the CAR hospital.

### Data analysis

We described inputs available in each hospital by computing the proportion of extended comprehensive PAC signal functions available in each hospital. Counts and proportions were used to describe the responses pattern of the health professionals in the KABP survey. Missing answers were classified as “don’t know” to follow a conservative approach. Midwifery/nursing assistants were not included in the analysis of medical knowledge and practices indicators of the KABP survey because they were not expected to know antibiotic and misoprostol regimens and to practice instrumental uterine evacuations. For the medical records review and the patients’ survey, participants characteristics (sociodemographic, reproductive, obstetrics) were summarized using the median with range for continuous variables or counts with proportion for categorical variables. Counts and proportions were also used to summarize the percentages of women with responses fulfilling each of the process and outcome quality-of-care indicators. We adopted a case study approach. Each site was unique and not representative of the population of hospitals in humanitarian settings. Therefore, even if we contrasted some characteristics and indicators, we did not do statistical testing. We calculated 95% confidence intervals (95% CI) of the proportions using the Clopper-Pearson exact method and performed analysis using Stata 16.0 software (College Station, Texas, USA).

## Results

### Inputs

Table 1 describes the knowledge, attitudes, and practices of health professionals in the two hospitals. 140 health professionals providing PAC in Nigeria (response rate: 99%) and 84 health professionals in CAR (response rate: 94%) responded to the KAPB survey. Among them, 92 were doctors, midwives, or nurses in Nigeria, and 78 in CAR. In both hospitals, around 90% of the health professionals reported having received training on PAC and almost 80% thought that PAC was every woman’s right. Nevertheless, doctors, midwives and nurses had gaps in certain knowledge and practices. While almost 70% answered the questions on misoprostol and antibiotics correctly in the CAR hospital, in Nigeria, this figure was 50% for the question on misoprostol and 21% for that on antibiotics. In addition, almost 20% of respondents in Nigeria and 35% in CAR reported using the inappropriate technology of dilatation and sharp curettage (D&C).

**Table 1:** Inputs indicators—human resources competencies in the Nigeria and CAR hospitals

Indicators	Nigeria hospital		CAR hospital	
	**n/N**	**Percentage** **%**	**n/N**	**Percentage** **%**
Self-reported that they were trained in PAC	123/140	87.9%	81/84	96.4%
Agreed that access to PAC is every woman’s right	111/140	79.3%	66/84	78.6%
Knew the recommended misoprostol posology to treat first trimester incomplete abortions	46/92	50.0%	54/78	69.2%
Knew the recommended antibiotic regimen to treat septic abortions	19/92	20.7%	53/78	68.0%
Reported using D&C (inappropriate technology) for instrumental uterine evacuations	18/92	19.6%	27/78	34.6%

*D&C* Dilatation and sharp Curettage

Table 2 describes the PAC signal functions available in each hospital. Of the 27 extended comprehensive PAC signal functions, 93% were available in the Nigerian and 96% in the CAR hospitals. The two signal functions which were missing in the Nigerian hospital were internet connection and contraception services 7 days per week (only provided 5 days/week). In the CAR hospital, the only signal function not fully available was an Intensive Care Unit, as the High Dependency Unit lacked mechanical ventilation systems.

**Table 2:** Inputs indicators—extended comprehensive post-abortion care signal functions in the Nigeria and CAR hospitals

	Nigeria hospital	CAR hospital
**Comprehensive PAC Signal functions**	**Indicator present** ^**d**^	**Indicator present** ^**d**^
Drugs, supplies, and services available for Post-Abortion Care		
Parenteral Uterotonics (at least 2 uterotonics with at least 1 parenteral available for PAC)	YES (1)	YES (1)
Removal of retention products (manual or electric vacuum aspiration for PAC)	YES (1)	YES (1)
Parenteral antibiotics	YES (1)	YES (1)
Intravenous fluids	YES (1)	YES (1)
Blood transfusion (with routine screening of donor blood for HIV, Hepatitis B, C and Syphilis)	YES (1)	YES (1)
Surgical laparotomy capability (including hysterectomy)	YES (1)	YES (1)
Drugs, supplies, and services available for Post-abortion Contraception		
3 + Modern short acting contraceptives (at least 3 methods)	YES (1)	YES (1)
1 + Modern long-acting reversible contraceptives (at least 1 method)	YES (1)	YES (1)
Contraception available 7/7	NO (0)	YES (1)
Infrastructure and Human Resources		
Facility open 24/7	YES (1)	YES (1)
1 + medical doctor on duty 24/7	YES (1)	YES (1)
3 + medical doctor registered and effectively working	YES (1)	YES (1)
TOTAL n/N, (%)	11/12(92%)	12/12(100%)
**Additional signal functions to fulfill the extended capability to provide comprehensive PAC**	**Indicator present**	**Indicator present**
Infrastructure		
Electricity available and functioning	YES (1)	YES (1)
Generator available and functioning	YES (1)	YES (1)
Refrigerator available and functioning	YES (1)	YES (1)
Email/internet available and functioning	NO (0)	YES (1)
Incinerator available and functioning	YES (1)	YES (1)
Water supply available and functioning	YES (1)	YES (1)
Sewerage system available and functioning	YES (1)	YES (1)
Referral capacity to refer patients if needed ^e^:		
Telephone/radio call available and functioning	YES (1)	YES (1)
Ambulance available and functioning	YES (1)	YES (1)
Guidelines, Equipment, and Human Resources		
Evidence based PAC guidelines available and accessible for clinicians ^b^	YES (1)	YES (1)
Clinical audits currently in use (regular mortality, morbidity and/or near-miss review)	YES (1)	YES (1)
Critical care unit available and functioning (ICU) ^c^	YES (1)—ICU	NO (0)—HDU
Ultrasound available and functioning	YES (1)	YES (1)
Biochemical/clinical laboratory available and functioning	YES (1)	YES (1)
Anesthetist capacity on duty 24/7	YES (1)	YES (1)
TOTAL n/N, (%)	14/15(93%)	14/15(97%)
**TOTAL EXTENDED CAPABILITY TO PROVIDE COMPREHENSIVE PAC** ^**a**^	25/27 (92.6%)	26/27 (96.3%)

^a^Sum of Comprehensive PAC signal functions + Additional signal functions

^b^Post Abortion Guidance/Clinical Handbook (MSF or WHO guidelines or evidence-based, locally adapted guidelines)

^c^As per the WHO near-miss approach definition [38], an intensive care unit (ICU) is a unit that provides 24-h medical supervision (including continuous vital signs monitoring), mechanical ventilation (including oxygen) and continuous vaso-active drugs. The High Dependency Unit (HDU) is a unit with all those characteristics except the mechanical ventilation

^d^Presence of a given indicator for a facility adds a score of one to the total category score for that facility

^e^Facility has the communication means (phone and radio) and the referral means (ambulance) to refer the patient in case of specific severe complications that are outside the management capacity of the facility

### Processes and outcomes

#### Population description

**Fig. 2:**
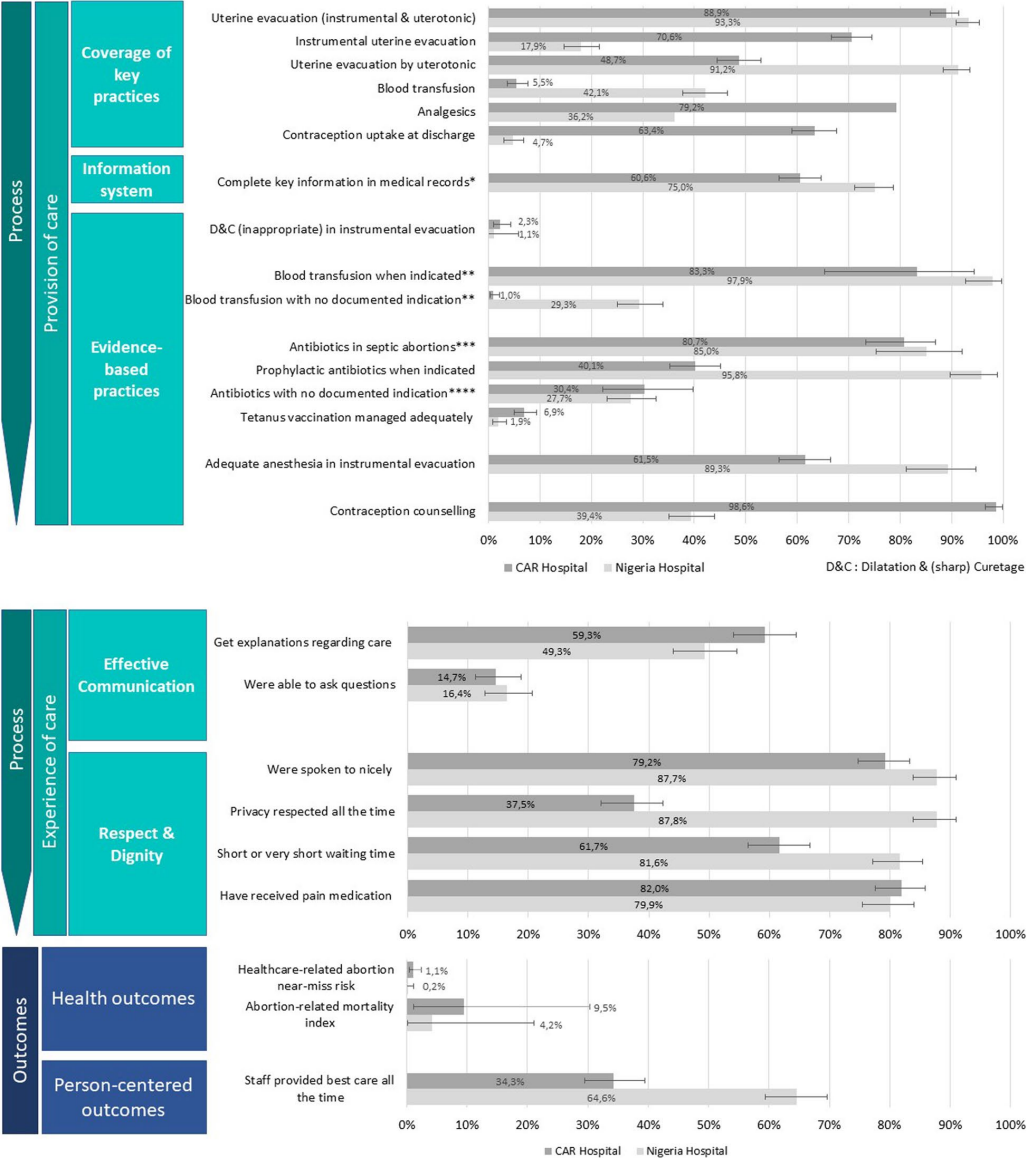
Process (provision & experience of care) & outcome indicators in the Nigeria and CAR hospitals *All the following key pieces of information are available in their medical record: estimate of gestational age, information on vital signs (temperature, systolic & diastolic blood pressures, heart rate, respiratory rate), abdominal examination, cervix examination, mental status, appearance at presentation & final diagnosis **A woman who had an indication of blood transfusion is defined by (MSF guidelines 2019 [37]) a woman with Hb ≤ 5 g/dl, even if there are no signs of decompensation or with Hb > 5 g/dl and < 7 g/dl if there are signs of decompensation (lowest SBP < = 90 mm Hg & pulse > = 100 b/min) or sickle cell disease or severe malaria or serious bacterial infection or pre-existing heart disease ***Septic abortions include uterine infections, generalized peritonitis, or severe systemic infections with genital origin ****No documented indication of (curative and prophylactic) antibiotics includes: no documented 1- infection, 2- instrumental/surgical procedure, 3- trauma/perforation (no evidence of cervix/vaginal mechanical injury at clinical examination, uterine perforation or other intra-abdominal perforation confirmed at laparotomy or at clinical examination), 4- notion of septic maneuver to induce abortion and 5- foreign body found in the vagina

Additional file 3 describes the study flow charts of the prospective medical records’ review and the patient survey in both study sites. A total of 520 women with abortion complications were included in the medical records’ review in the Nigerian and 548 in the CAR hospital. Among them, 360 (69%) and 362 (66%) participated in the patient survey in Nigeria and CAR. Most of the women who were not included did not stay overnight (not eligible for the quantitative survey) or were discharged before the interview. Some of the sociodemographic, reproductive, and obstetric characteristics of the women in the two hospitals were different, as indicated in Additional file 4. Women were older in the Nigerian hospital where the majority (82%) were married, while in the CAR hospital, most women were unmarried (70%). Women of the Nigerian hospital had a much lower education level (62% had no formal education) than women of the CAR hospital (72% reached at least a secondary school level). In addition, among all included women, 62% presented in their second trimester of pregnancy in the Nigerian hospital compared to 33% in the CAR hospital. More than half presented with severe complications; and 18.7% had septic abortions in Nigeria and 27% in CAR. Figure 2 shows the quality of PAC process and outcomes indicators for each hospital; these are further detailed in Additional file 5.

#### Process—provision of care

Only 60% of medical records in the CAR hospital and 75% in the Nigerian hospital had complete medical information. Almost all women included in the medical records review had a uterine evacuation (93% in Nigeria, 89% in CAR), but practices varied across hospitals. In Nigeria, uterotonics were most often used (91%), while in CAR, instrumental evacuations were prevalent (71%). In both hospitals, very few women received the non-recommended method of D&C (1.1% in Nigeria and 2.3% in CAR). Most women in need of blood transfusion received it (98% in Nigeria and 83% in CAR). However, in the Nigerian hospital, 29% of women received some blood transfusion when there was no documented indication (versus 1% in CAR). In both hospitals, more than 80% of women with septic abortion signs received antibiotics but antibiotics were also given to about 30% of patients with no documented indication. Although 96% of women in Nigeria received prophylactic antibiotics when indicated, only 40% did in CAR. In addition, in both hospitals, very few women had their tetanus immunization status checked and managed (2% of all Nigerian women and 7% of all CAR women). In terms of pain management, 36% of women received analgesics in the Nigerian hospital and 79% in the CAR hospital. Nevertheless, only 61% received a recommended method of anesthesia during instrumental uterine evacuation in the CAR hospital, compared with 89% in the Nigerian hospital. Virtually all discharged women in CAR (99%) received contraceptive counselling, but only 39% in Nigeria. This resulted in 63% of women in CAR being discharged with a contraceptive method compared to 5% of women in Nigeria.

#### Process—experience of care

Women generally reported good experience of respect and preservation of dignity in the Nigerian hospital. Experiences in this domain were more mixed in the CAR hospital. Most women reported that they were spoken to nicely (88% in Nigeria and 79% in CAR), that they received pain medication (80% in Nigeria and 82% in CAR) and that their waiting time was short or very short (82% in Nigeria and 62% in CAR). While 88% of women in Nigeria reported that their privacy was respected all the time during physical examinations, only 37% of the CAR women did. Women reported poor experience of care regarding communication; 49% in Nigeria and 59% in CAR said they were given explanations about their care and around 15% of women in both hospitals said they felt able to ask questions during examination and treatment.

#### Outcomes

The facility-based risk of healthcare-related abortion-near-miss was at 0.2% in Nigeria and 1.1% in CAR. Only 65% of women in Nigeria and 34% in CAR reported that the staff provided them best care all the time.

## Discussion

Our comprehensive assessment of the quality of PAC suggested that overall, the two hospitals included in this study provided lifesaving PAC. The risk of healthcare-related near-miss was low (< 2%), as was the mortality index, when compared to other facilities in Africa (9.5% in the CAR hospital and 4.2% in the Nigerian hospital versus 18.3% in the WHO-MCS-A [47]). However, we noted a possible overuse of antibiotics and blood transfusion, suggesting overmedicalization and inefficient use of resources. Further, women reported mixed experiences with the quality of care provided.

The low risk of healthcare-related near-miss can likely be explained by the adequate availability of healthcare inputs permitting the proper implementation of evidence-based practices. Almost all health professionals had received PAC training and almost all comprehensive and extended PAC signal functions were available in both hospitals, which has not been the case for many other African referral hospitals studied in more stable contexts [40, 48]. Some of the key evidence-based practices were correctly implemented in both hospitals including the provision of blood transfusion when indicated, antibiotics administered to patients with septic abortions and use of appropriate technology to evacuate the uterus. Less than 2.5% of patients received the non-recommended and less safe D&C technology when having an instrumental uterine evacuation. This estimate is lower than in other studies in Africa (ranging from 8 to 100% [19, 47, 49–54]) but is similar to levels found in facilities supported by international organizations [24, 55]. Nevertheless, this result is contradictory with the fact that a fifth to a third of clinicians reported using D&C to evacuate the uterus respectively in the Nigerian and CAR hospitals. This contradiction might be explained by a possible confusion of the definition of D&C by the clinicians answering the KAPB questionnaire, especially in CAR where the expression “curettage” is often used to name different types of instrumental uterine evacuation, including the recommended MVA [56].

Our results also suggest inadequate knowledge and use of antibiotics among healthcare providers in both settings. Although most women with septic abortion received antibiotics, they may not have received the recommended regimen. In fact, the KAPB survey identified insufficient knowledge among physicians, midwives, and nurses on this topic, particularly in the Nigerian hospital. In contrast, prophylactic antibiotic therapy for the prevention of post-instrumental uterine evacuation infections seems to be well implemented in the Nigerian hospital, unlike in the CAR hospital. This preventative intervention is even more important in these contexts of restrictive abortion laws in both countries where women may have had unsafe instrumental abortions [26]. Moreover, as found in other African studies [57, 58], we identified some over-prescription of antibiotics in both settings, even though some of these prescriptions might have been justified but not documented in the patient file. Thus, although a large proportion of healthcare professionals reported having been trained in PAC, it is possible that this training was not recent and/or did not cover antibiotic therapy.

In addition to an overprescription of antibiotics, the possible overuse of blood transfusion in the Nigerian hospital or the high use of instrumental uterine evacuation in the CAR hospital could suggest “overmedicalization” for some patients. It might be due to provider preference and practice or organizational constraints, rather than evidence-based recommendations [59]. In CAR, the choice of the faster method to evacuate the uterus (MVA) might be due to the high bed occupancy rate. The fact that only 15% of patients felt they were able to ask questions about their treatment suggest that patients’ preference might not have always been asked or taken into consideration. On the other hand, in the Nigerian hospital, some women might have had an undocumented indication of blood transfusion. Alternatively, the prescription of blood transfusions outside the guidelines’ indications may suggest the need to adapt these recommendations to the context. Indeed, women of reproductive age in this fragile State of Jigawa have some of the worst nutritional and anemia indicators in the country [26, 60]. The fact that resources in this hospital are not as restricted as in other hospitals not supported by international organizations [57] may have enabled clinicians to adapt their practice to the specificities of women in this state.

The provision of contraceptive services was found to be insufficient in the Nigerian hospital compared to the CAR facility or other African hospitals studies [57, 61, 62]. One reason for this may be a lack of coordination of PAC services with the contraceptive services provided in another hospital department that was not opened 7/7. The limited women’s autonomy in accessing contraception in this region may also contribute to this result [63].

In both study hospitals, around 80% of women reported having received pain medications. However, pain management was not optimal according to the medical records. In the CAR hospital, anesthesia was recorded as provided only to six out of ten women undergoing instrumental uterine evacuation, despite paracervical block being a part of the standard protocol. In contrast, in the Nigerian hospital, while anesthesia was recorded as provided almost routinely in instrumental evacuation, only 36% of patients received analgesics according to the medical records’ review. The discrepancy between the reported experience by women and the medical records review may be explained by a lack of documentation of analgesic in the medical files, a desirability bias (women not daring to say they did not receive analgesic), or women misunderstandings of the treatment provided. This latter hypothesis is supported by the fact that the lack of effective provider-patient communication was the most important gap identified in the patients’ survey. Providers may not have time to give the chance to women to ask questions. More generally, they may be guided by implicit bias in the provision of person-centered care, thinking, for example, that these women are unlikely to have questions or understand explanations or to be able to decide which care options to choose. This may partly explain the low proportions of women reporting that caregivers provided them with the best care all the time, especially in the CAR hospital. In addition, this lower satisfaction in CAR may be helped by the fact that more women in CAR than in Nigeria reported a lack of respect for their privacy and long waiting times. Yet, evidence shows that poor communication, long waiting times and lack of privacy in hospitals may be a significant barrier in women’s satisfaction with care and adherence to treatment [64], also preventing women’s engagement in their own care. The differences in patients’ characteristics between the two settings may also explain this difference in the person-centered outcomes. They could affect participants responses, with the Nigerian women being more likely to be older, married, with less education and more severe complications than the CAR women. Individual experiences of care are highly subjective variables [65]. Differences between the two settings might be due to different levels of patients’ understanding and expectations of quality according to their characteristics or different social norms [42].

Overall, the quality of care provided in these two hospitals of humanitarian settings can partly be explained by the important support of MSF to the two facilities in terms of provision of equipment, medication, staffing, continuous training, supervision, and availability of medical protocols. Other research in humanitarian contexts assessing the impact of NGO interventions found important improvement in some quality indicators [24, 55]. This suggests that even in such challenging contexts, providing and improving quality of PAC inside health facilities is feasible and that some of the potential barriers linked to fragility or insecurity can be overcome.

### Strengths and limitations

Our comprehensive assessment examined almost all the quality domains included in the WHO framework [10]. This represents a clear added-value compared with other quality assessments of PAC in humanitarian settings and/or low-and-middle-income countries which generally assess one to three domains, mainly hospitals’ essential physical and human resources [18, 39, 40, 48, 66–69], coverage of key practices [23–25, 69, 70] or patients’ experience of care [21, 23, 25, 70–73]. The inclusion of a full set of indicators assessing the implementation of evidence-based practices and one indicator assessing the information system is a clear asset, since most of these indicators are rarely included in PAC assessments [19, 69].

We applied a more robust health outcome measure than the mortality index which has been used in other studies [38]. A limitation of this indicator is that it does not exclude inevitable deaths from the estimates and therefore doesn’t accurately reflect the outcome of the care provided in the health facility. As we have done in our study, we recommend that future quality-of-care research uses the risk of healthcare-related near-miss as their outcome indicator [46]. This indicator corrects for the flaw of the mortality index because it measures the worsening of the state of the women after 24h and eliminates most inevitable deaths or near-miss that are happening in the first 24h after presentation and for which the responsibility of the quality of care provided in the facility is difficult to determine.

The use of multiple sources of information to measure the indicators increases the robustness of the assessment by considering and triangulating different points of view. The analysis of the similarities and discontinuities between the inputs, process and outcome indicators allowed to strengthen our understanding of the issues identified, enabling field-oriented recommendations to be formulated. This suggests that this approach could be applied in all types of hospital settings, whether stable or humanitarian, and supported or not by NGO.

Nevertheless, our study faced several limitations. Because the insecurity prevailing in the areas obliged us to collect data in only one referral hospital in each setting, our results cannot be generalized to other hospitals of the targeted areas, regions, and countries, nor to hospitals of humanitarian settings. And this assessment only focused on the quality of care provided to women with more serious complications. Women with no or very mild complications were not included because they did not have medical records (Additional file 3) and experience-of-care indicators were only measured in women who stayed at least overnight.

In addition, only few indicators per domain of the WHO framework were selected, improving the assessment feasibility but limiting its content validity. While most of these indicators have been used in other studies led in Sub-Saharan Africa, and some have been extracted from validated questionnaires [42, 43], they have not been selected through a structured consensus-based method [34] that could have included end-users, nor have they been comprehensively validated as a tool.

Although emotional support is a key dimension of experience of care, its indicators were removed from our assessment because questions were identified as ambiguous [44]. Furthermore, even if rigorous confidentiality procedures have been implemented and interviewers were independent from health providers, some memory and desirability biases can remain in the providers’ and women’s answers to surveys, limiting the validity of the results, especially in a subject like abortion, which is prone to stigma.

While the same prospective methodologies were used to collect data in the two hospitals, and the same management guidelines and standardized medical records were applied, some documentation completeness and differences in the patients’ files may have remained. This may limit the validity and comparability of the evidence-based practices and health outcomes indicators.

### Implications for policies, practices and research

To improve PAC quality in all hospitals including in humanitarian settings, efforts should be maintained to completely abandon D&C and ensure a continuous use of the appropriate technology to evacuate the uterus. Practitioners’ continuous training, and regular antibiotic stewardships should be implemented to promote adequate and rational use of antibiotics to better prevent and treat infections and to avoid antimicrobial resistance in the longer term [74]. Post-abortion contraception should be provided at the same time and location as clinical treatment for complications to increase the uptake of contraceptive methods by women and thus protects them against the risk of future unintended pregnancies [20, 48, 75]. In addition, there is an urgent need to initiate strategies to enhance communication with patients about their condition, care, and post-abortion contraception in a supportive, empathetic, and nonjudgmental attitude as well as ensuring short waiting times, patients’ privacy in examinations and adequate pain management. To improve women's satisfaction with care and increase the likelihood of timely contraception uptake in the absence of pregnancy desire, practitioners should use educational protocols in PAC, using job aids and leaflets to provide information about women's treatment, postabortion fertility, and contraception [73, 75–78]. They also should conduct workshops to clarify values and attitudes about abortion to mitigate stigma [79].

Assessing PAC quality should be regularly implemented as part of maternal health care quality processes in all hospitals including in humanitarian settings. Our assessment done in the context of a research study requires the use of different sources of data which can be challenging to collect routinely. Researchers should do more work to validate short sets of indicators for routine assessment which for example could include the following minimum package: PAC signal functions check list, indicators of the quality of the documentation, the evidence-based practices and the healthcare outcome assessed retrospectively in a random sample of patients’ medical records and, indicators of experience of care and the person-centered outcome assessed by short patients exit surveys. Finally, as WHO recommends that quality care should be equitable [10], further research should be done to assess if PAC quality indicators differ according to socio-demographic backgrounds.

## Conclusion

Our comprehensive assessment of the quality of PAC in two hospitals in humanitarian settings showed that providing lifesaving PAC was feasible in such contexts, accounting for the low risk of near-miss events happening 24 h after presentation. Both hospitals had almost all the equipment and human resources needed to provide PAC. Most health providers followed evidence-based recommendations for the provision of blood transfusion when indicated and for the use of appropriate technology to evacuate the uterus. Nevertheless, we identified key areas for improvement over a broad range of indicators. Although most providers had received training on PAC, gaps remained in knowledge and application of guidelines, particularly regarding uterotonics, antibiotics and pain management. Both hospitals would benefit from the implementation of continuous training on these topics and from setting-up antibiotic stewardships to prevent nosocomial infections as well as antibiotic-resistance. In addition, systematic integration of contraceptive counseling and provision into PAC services would help reduce women’s unmet contraceptive needs. Finally, it is urgent to improve the patient-centeredness of PAC, ensuring privacy during all examinations, enhancing patient-provider communication and engaging women to participate in the decision regarding their own care.

### Supplementary Information

Additional file 1. Fragility, conflict, and natural disaster exposures of the surrounding areas of the 2 participating hospitals.

Additional file 2. Quality of Post-Abortion Care quality measures and indicators measured in AMoCo study.

Additional file 3. Study flow charts for abortion complications included in the medical records reviews and the quantitative surveys in the Nigerian and CAR study hospitals.

Additional file 4. Characteristics of women with abortion complications in Nigeria and CAR study hospitals.

Additional file 5. Process (provision & experience of care) & outcome indicators measuring the quality of post-abortion care provided in the Nigerian and CAR hospitals.

## Data Availability

The AMoCo study protocol is available in the MSF science portal: https://scienceportal.msf.org/assets/7660. The dataset collected during the study, including deidentified participant data, data dictionary and additional related documents (data collection tools, manual of procedures, interviewers guide, standard operating procedures) are available from the corresponding author or dpco@epicentre.msf.org on reasonable request, following MSF's data sharing policy which ensures that data will be available upon request to interested researchers while addressing all security, legal, and ethical concerns, especially for sensitive subjects like abortion in vulnerable populations.

## Abbreviations

AMoCo: Abortion-related Morbidity and mortality in fragile and Conflict-affected settings (study)
CAR: Central African Republic
D&C: Dilatation and sharp Curettage
KAPB: Knowledge Attitudes Practices and Behavior
MNHC: Maternal and Newborn Health Care
MRR: Prospective Medical Records Review
MSF: Médecins Sans Frontières
MVA: Manual Vacuum Aspiration
NGO: Non-Governmental Organization
PAC: Post-Abortion Care
WHO: World Health Organization
WHO-MCS-A: World Health Organization Multi-Country Study on Abortion
