# Supplementary material for: Assessing post-abortion care using the WHO quality of care framework for maternal and newborn health: a cross-sectional study in two African hospitals in humanitarian settings
Source: Reprod Health. 2024 Aug 5;21:114. doi: 10.1186/s12978-024-01835-9 (PMC11299292; doi:10.1186/s12978-024-01835-9)
Supplement: Supplementary file 1 — Additional file 1. Fragility, conflict, and natural disaster exposures of the surrounding areas of the 2 participating hospitals. [file 12978_2024_1835_MOESM1_ESM.pdf]

<u>Additional file 1</u>: Fragility, conflict, and natural disaster exposures of the areas of the 2 participating hospitals

Study site	Surrounding Areas	Fragile State Index classification in 2019 and 2020 (from the most fragile to the most stable over 178 states)(8,9)	Conflict-related events /month during the duration of the participants' pregnancies (1,2)	Conflict-related deaths/year/100 000 persons during the duration of the participants' pregnancies* (1,2)	Natural disaster events during the duration of the participant's pregnancies(3)	Flooding number of events and duration during the duration of the participant's pregnancies(4)
CAR study site	**Bangui**	6th (high alert)	4.3	**5.7***	Flooding	5 flooding episodes<br>Mean duration: 1.1 hours/month
Nigeria study site	**Jigawa State**	14th (alert)	1.3	0.1	**Flooding<br>Lassa fever**	**14 flooding episodes<br>Mean duration: 64.1 hours/month**
	Yobe State		3.8	2.5*	Flooding	20 flooding episodes<br>Mean duration: 11.6 hours/month
	Katsina State		13.4	2.2*	Flooding<br>Yellow fever	12 flooding episodes<br>Mean duration: 18.3 hours/month
	Bauchi State		1.2	0.1	Flooding<br>Lassa fever<br>Yellow fever	16 flooding episodes<br>Mean duration: 29 hours/month
	Kano State		1.5	0.02	Flooding<br>Lassa fever<br>Yellow fever	33 flooding episodes<br>Mean duration: 59.8 hours/month

* Setting with medium-intensity conflict according to the World Bank (2 to 10 deaths/year/100 000 population)(7).

<u>Methodology used to identify the study settings' exposure to conflict and natural disaster:</u>

To describe the exposure to conflict and natural disaster in both settings with comparable data, we extracted data from international independent databases. For each study context (Bangui, Jigawa State and its neighbors States: Yobe, Katsina, Bauchi and Kano), we extracted 1) the number of conflict events in the Armed Conflict Location and Event Data (ACLED) database(1,2); 2) the natural disaster events in the international disaster database(3); 3) the number and duration of flooding events in the global flood monitor(4). The ACLED database collects location, actors, fatalities and date information on all reported political violence and protest events daily(1,2). The international disaster database lists the disasters that happened during defined periods(3). The global flood monitor detects, in real-time, regions with enhanced flood-related Twitter activity and classifies these as flood events(4). The data were extracted for the duration of the pregnancies of the women included in the study. Population data of Bangui(5) and the Northern Nigeria States(6) were also used. The mean number of hours of flooding/months, number of conflict-events/month and number of deaths/year/100 000 persons were computed using Excel software. Settings with 2 to 10 deaths/year/100 000 population are classified as setting with medium-intensity conflict by the World Bank(7).

Study settings' exposure to conflict and natural disaster

During the duration of the pregnancies of the women included in the CAR study site (April 2019 to January 2020), Bangui suffered an average of 4.3 conflict-related events/month causing 5.7 deaths/year/100 000 persons classifying them as medium-intensity conflict-affected settings(7). In addition, during the same period, Bangui suffered from 5 episodes of floods that last an average of 1,1 hour/month. (cf. table appendix 5)

During the duration of the pregnancies of the women included in the Nigeria study site (August 2019 to July 2021), Jigawa State endured an average of 1.3 conflict-related events/month causing 0.1 deaths/year/100 000 persons. The neighboring States of Yobe and Katsina suffered respectively an average of 3.8 and 13.4 conflict-related events/month causing respectively 2.5 and 2.2 deaths/year/100 000 population, classifying them as medium-intensity conflict-affected settings(7). While Jigawa State experienced less conflict-related events than Bangui, it had to deal with more natural disaster events. Between August 2019 and July 2021, it faced one Lassa fever outbreak and 14 episodes of intense flooding that last an average of 64,1 hours/month. (cf. table appendix 5)

References:

1. Raleigh C, Linke A, Hegre H, Karlsen J. Introducing ACLED: An Armed Conflict Location and Event Dataset. J Peace Res [Internet]. 2010 [cited 2022 Mar 3];47(1):651–60. Available from: https://www.jstor.org/stable/20798933
2. ACLED. Armed Conflict Location & Event Data - Data Export Tool [Internet]. 2022 [cited 2022 Mar 3]. Available from: https://acleddata.com/data-export-tool/
3. CRED/UCLouvain. EM-DAT, The International Disaster Database [Internet]. 2021 [cited 2022 Feb 3]. Available from: https://www.emdat.be/
4. de Bruijn JA, de Moel H, Jongman B, de Ruiter MC, Wagemaker J, Aerts JCJH. A global database of historic and real-time flood events based on social media. Sci Data 2019 61 [Internet]. 2019 Dec 9 [cited 2022 Mar 3];6(1):1–12. Available from: https://www.nature.com/articles/s41597-019-0326-9
5. Direction Générale de la statistique des études économiques et sociales - République Centrafricaine. Recensement général de la population et de l'habitation [Internet]. Bangui, République Centrafricaine; 2003. Available from: https://icasees.org/nada/index.php/catalog/40
6. National Population Commission (NPC) [Nigeria], ICF. Nigeria Demographic Health Survey 2018 [Internet]. Abuja, Nigeria, and Rockville, Maryland, USA; 2019. p. 748. Available from: https://dhsprogram.com/publications/publication-fr359-dhs-final-reports.cfm
7. The World Bank. Revised Classification of Fragility and Conflict Situations for World Bank Group Engagement [Internet]. 2019. p. 1–3. Available from: https://peacekeeping.un.org/en/where-we-operate.
8. Fund For Peace. Fragile State Index Annual Report 2020. Geneva: Fund for Peace; 2020.
9. Fund For Peace. Fragile States Index Annual Report 2019 [Internet]. 2019. Available from: https://fragilestatesindex.org/2019/04/07/fragile-states-index-2019-annual-report/
