# Supplementary material for: Assessing post-abortion care using the WHO quality of care framework for maternal and newborn health: a cross-sectional study in two African hospitals in humanitarian settings
Source: Reprod Health. 2024 Aug 5;21:114. doi: 10.1186/s12978-024-01835-9 (PMC11299292; doi:10.1186/s12978-024-01835-9)
Supplement: Supplementary file 2 — Additional file 2. Quality of Post-Abortion Care quality measures and indicators measured in AMoCo study. [file 12978_2024_1835_MOESM2_ESM.pdf]

**Additional file 2:** Post-Abortion Care quality measures and indicators measured in AMoCo study

| **Donabedian** [1] | **Domains from WHO QoC framework for MNHC** [2] | **Quality measures and corresponding indicators measured in AMoCo study** | **Targets** | **Data source** |
|---|---|---|---|---|
| **1-Structure/Inputs** | Competent human resources | **Adequate knowledge, attitudes & practices of health professionals**<br>*Training & positive attitudes in PAC*<br>Percentage of doctors, medical officers, midwives/nurses and midwifery/nurse assistant who are providing post-abortion care and who [a]:<br>- have been trained in Post-Abortion Care<br>- agreed that access to post-abortion care is every woman's right<br>*Correct knowledge in management of key complications*<br>Percentage of doctors, medical officers, midwives/nurses who are providing post-abortion care and who [a]:<br>- knew the recommended misoprostol regimen to treat first trimester incomplete abortions<br>- knew the recommended antibiotic regimen to treat septic abortions<br>- reported using D&C (non-recommended inappropriate technology) when treating incomplete abortion by instrumental uterine evacuation | 100 %<br>100 %<br>100 %<br>100 %<br>0% | Knowledge, Attitude Practice Behavior survey – Health professionals [a] |
| | Essential physical resources<br>Functional referral systems | **Extended comprehensive post-abortion care signal functions** *(Trained medical professionals on duty 24/7, infrastructure, Drugs, supplies & services for PAC and for contraception available)*:<br>Percentage of the extended comprehensive post-abortion care signal functions (n=27) available in the facility (cf. table 2) [3, 4] | 100 % | Rapid Facility Assessment |
| **2- Processes** | **Provision of care** – Coverage of key practices | **Coverage of key practices**<br>- *Uterine evacuation*: Percentage of women who received<br>• instrumental uterine evacuation<br>• uterine evacuation by uterotonics<br>- *Key interventions for severe complications:* Percentage of women who received<br>• blood transfusion<br>• IV fluids<br>• Intensive Care Unit admission<br>• hysterectomies<br>- *Pain management:* Percentage of women who received analgesics<br>- *Contraception uptake:* Percentage of women who received Contraception at discharge | N/A | Prospective Medical Record Review |
| | **Provision of care** – Actionable information system | **Information system**<br>*Completeness of clinical medical records on key medical information:*<br>Percentage of women with complete information in their medical records on key medical information: gestational age, vital signs, abdominal examination, cervix examination (proxy for gynecological exam), mental status and appearance at presentation & final diagnosis | 100 % | |

| Donabedian [1] | Domains from WHO QoC framework for MNHC [2] | | Quality measures and corresponding indicators measured in AMoCo study | Targets | Data source |
|---|---|---|---|---|---|
| 2- Processes (to be continued) | Provision of care (to be continued) | Evidence based practices for routine care and management of complications | **Application of post-abortion care evidence-based clinical guidelines (practices)** [5]: | | Prospective Medical Record Review |
| | | | *Appropriate (preventative or curative) treatment for* | | |
| | | | - *Uterine evacuation:* Percentage of women undergoing Dilatation & sharp Curettage when having an instrumental uterine evacuation (inappropriate) | 0% | |
| | | | - *Hemorrhage*: Percentage of women | | |
| | | | • receiving blood transfusion when indicated [b] | 100 % | |
| | | | • receiving blood transfusion when there is no reported indication [b] (inappropriate) | 0% | |
| | | | - *Infections*: Percentage of women | | |
| | | | • with septic abortion [c] receiving curative antibiotics | 100 % | |
| | | | • receiving prophylactic antibiotics when having an instrumental/surgical intervention (instrumental uterine evacuation, hysterectomy, laparotomy, laparoscopy) | 100 % | |
| | | | • receiving antibiotics when there is no reported indication [d] (inappropriate) | 0% | |
| | | | • with tetanus vaccination status assessed & adequately managed | 100 % | |
| | | | - *Anemia*: Percentage of women with anemia who were prescribed Iron/Folic acid at discharge | | |
| | | | - *Pain management*: Percentage of women having instrumental uterine evacuation who received adequate anesthesia (para cervical block, spinal anesthesia, general anesthesia) | 100 % | |
| | | | *Contraception counselling:* Percentage of women having been counselled about contraception. | 100 % | |
| | Experience of care | | **Reported experience of post-abortion care:** Percentage of women who reported that [e] | | Quantitative patient survey [e] |
| | | Effective communication | - they *were given explanations* regarding care (UCSF PCMC scale & WHO-MCS-A question) | 100 % | |
| | | | - they *were able to ask questions* during treatment (UCSF PCMC scale & WHO-MCS-A question) | 100 % | |
| | | Respect and preservation of dignity | - they *were spoken to nicely* (WHO-MCS-A question) | 100 % | |
| | | | - they *received pain medication* (WHO-MCS-A question) | 100 % | |
| | | | - their *privacy was respected* all the time during physical examinations (UCSF PCMC scale) | 100 % | |
| | | | - they had *very short or short waiting time* to see a provider (UCSF PCMC scale) | 100 % | |
| | | Emotional support | - they were *asked about their feelings* (UCSF PCMC scale) | 100 % | |
| | | | - they *have been supported* by doctors or nurses about *their anxieties* and fears (UCSF PCMC scale) | 100 % | |

| **Donabedian** [1] | **Domains from WHO QoC framework for MNHC** [2] | | **Quality measures and corresponding indicators measured in AMoCo study** | **Targets** | **Data source** |
|---|---|---|---|---|---|
| **3- Outcomes** | **Outcomes** | Person-centered outcomes | *Good overall quality of the post-abortion care provided as perceived by the patient:*<br>Percentage of women who reported that staff provided <u>best care all the time</u> (UCSF PCMC scale) [e] | 100% | Quantitative patient survey[e] |
| | | Abortion-related health outcomes | *Low*<br>- *Abortion-related Mortality Index*: number of deaths/number of severe maternal outcome (SMO = near-miss cases† and deaths)<br>- *Risk of Healthcare-related abortion near-miss†:* number of women with Near-miss† happening >= 24h after presentation/total number of women presenting for abortion complications | The lower, the better | Prospective Medical Record Review |

[a] Questions included in the KAP survey questionnaire among health professionals providing post-abortion care:

- Did your medical/clinical officer/midwifery/nursing school training include any content on post-abortion care? (yes/no)
- Access to post abortion care services is every woman's right (Agree/Neutral, Unsure/Disagree)
- According to the guidelines used in this health facility, how many micrograms of misoprostol are recommended to treat incomplete abortion in the 1st trimester with a first single oral dose?
  - Correct answer: 600 micrograms
- According to the guidelines used in this health facility, what are the antibiotics to be given to treat septic abortion?
  - Correct answer: Amoxicillin/Clavulanic acid or Amoxicillin + metronidazole or Amoxicillin/Clavulanic acid + Gentamycin
- Do you personally provide post-abortion care with Dilatation & Curettage at this facility? (yes/no)

[b] a woman who needed a transfusion is defined by (MSF guidelines 2019[5]) a woman with:
- Hb ≤ 5 g/dl, even if there are no signs of decompensation
- Hb > 5 g/dl and < 7 g/dl if there are signs of decompensation (lowest SBP <= 90 mm Hg & pulse >= 100 b/min) or sickle cell disease or severe malaria or serious bacterial infection or pre-existing heart disease.

[c] Septic abortions include uterine infections, generalized peritonitis or severe systemic infections with genital origin and exclude extra-genital infections (malaria, urinary infections, etc.)

[d] No indication of antibiotics includes: no documented infection, no instrumental/surgical procedure, no trauma/perforation (no evidence of cervix/vaginal mechanical injury at clinical examination, uterine perforation or other intra-abdominal perforation confirmed at laparotomy or at clinical examination), no notion of septic maneuver to induce abortion and no foreign body found in the vagina.

[e] Questions included in the quantitative survey questionnaire among women who stayed at least overnight with abortion complications:

- During your stay at this hospital, were you given explanations regarding your care and treatment? (yes/no)
- Were you able to ask questions during the examination and treatment? (yes/no)
- Were you spoken to nicely? (yes/no)
- Did you receive pain medications during your hospital stay? (yes/no)
- During physical examinations (when the doctor was looking at private parts of your body) were you covered up (or given privacy in such a way only the doctor and nurses examining you could see you)? (Never/A few times/Most of the time/All the time/ Not examined)
- How do you feel about the amount of time you waited (to see a healthcare provider)? Would you say it was: (Very short/Somewhat short/Somewhat long/Very long)
- Did the doctors and nurses at the facility talk to you about how you were feeling? (yes/no)
- Did the doctors, nurses or other staff at the facility support your anxieties and fears? (yes/no)

- Did you feel the doctors, nurses or other staff at the facility took the best care of you? (Never/A few times/Most of the time/All the time)

† Near- miss cases: women with organ dysfunction of either one or more of the following: cardiovascular, respiratory, renal, coagulation, hepatic, neurological or uterine dysfunction using WHO near-miss criteria[6, 7].

SMO: Severe Maternal Outcome = Near-miss cases + Deaths

QoC: Quality of Care

WHO-MCS-A: World Health Organization Multi-Countries Study on Abortion[8]

UCSF PCMC scale: Person-Centered Maternity Care scale (University of California San Francisco)[9]

## References:


1. Donabedian A. Evaluating the Quality of Medical Care. Milbank Q. 2005;83:691.

2. WHO. Standards for improving quality of maternal and newborn care in health facilities. Who. 2016;:73.

3. Compaoré R, Mehrtash H, Calvert C, Qureshi Z, Bello FA, Baguiya A, et al. Health facilities' capability to provide comprehensive postabortion care in Sub-Saharan Africa: Evidence from a cross-sectional survey across 210 high-volume facilities. Int J Gynecol Obstet. 2022;156:7–19.

4. Cresswell JA, Owolabi OO, Chelwa N, Dennis ML, Gabrysch S, Vwalika B, et al. Does supportive legislation guarantee access to pregnancy termination and postabortion care services? Findings from a facility census in central Province, Zambia. BMJ Glob Heal. 2018;3.

5. MSF. Essential Obstetric and Newborn care : Practical guide for midwives, doctors with obstetrics training and health care personnel who deal with obstetric emergencies. 2019.

6. Qureshi Z, Mehrtash H, Kouanda S, Griffin S, Filippi V, Govule P, et al. Understanding abortion-related complications in health facilities: results from WHO multicountry survey on abortion (MCS-A) across 11 sub-Saharan African countries. BMJ Glob Heal. 2021;6:e003702.

7. World Health Organization, Organization WH. Evaluating the quality of care for severe pregnancy complications: the WHO near-miss approach for maternal health. Geneva: World Health Organization; 2011.

8. Kim CR, Tunçalp Ö, Ganatra B, Gülmezoglu AM. WHO Multi-Country Survey on Abortion-related Morbidity and Mortality in Health Facilities: study protocol. BMJ Glob Heal. 2016;1:e000113.

9. Afulani PA, Diamond-Smith N, Golub G, Sudhinaraset M. Development of a tool to measure person-centered maternity care in developing settings: Validation in a rural and urban Kenyan population. Reprod Health. 2017;14:1–18.
