# Supplementary material for: Assessing post-abortion care using the WHO quality of care framework for maternal and newborn health: a cross-sectional study in two African hospitals in humanitarian settings
Source: Reprod Health. 2024 Aug 5;21:114. doi: 10.1186/s12978-024-01835-9 (PMC11299292; doi:10.1186/s12978-024-01835-9)
Supplement: Supplementary file 3 — Additional file 3. Study flow charts for abortion complications included in the medical records reviews and the quantitative surveys in the Nigerian and CAR study hospitals. [file 12978_2024_1835_MOESM3_ESM.pdf]

Additional file 3: Study flow charts for abortion complications included in the medical records reviews and the quantitative surveys in the Nigerian and CAR study hospitals.

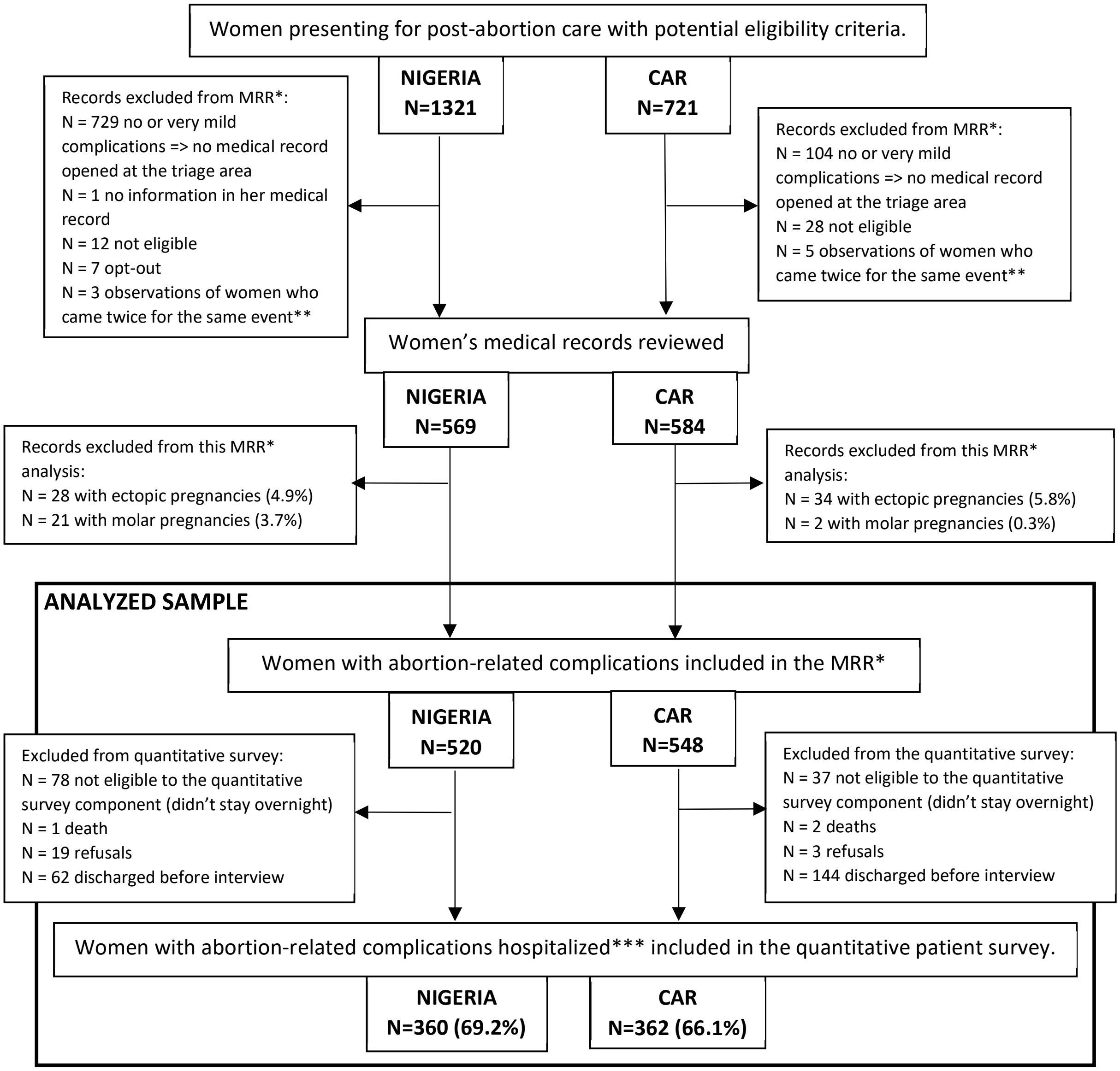

*MRR: Prospective Medical Records' Review component
** The observation with the most severe complication was kept
*** Hospitalized: who stayed at least overnight
