# Supplementary material for: Assessing post-abortion care using the WHO quality of care framework for maternal and newborn health: a cross-sectional study in two African hospitals in humanitarian settings
Source: Reprod Health. 2024 Aug 5;21:114. doi: 10.1186/s12978-024-01835-9 (PMC11299292; doi:10.1186/s12978-024-01835-9)
Supplement: Supplementary file 4 — Additional file 4. Characteristics of women with abortion complications in Nigeria and CAR study hospitals. [file 12978_2024_1835_MOESM4_ESM.pdf]

<u>Additional file 4</u>: Characteristics of women with abortion complications in Nigeria and CAR study hospitals

	Nigeria			CAR		
	**n**	**%**	**(95%CI)**	**n**	**%**	**(95%CI)**
**<u>Socio-demographic and admissions characteristics</u>**						
**Age group (years)**	**N=520**			**N= 546** (missing: 2)		
Median (range)	*27 (14-50)*			*24 (13-47)*		
≤19	87	16.7	(13.6-20.2)	142	26.0	(22.4-29.9)
20-29	225	43.3	(39.0-47.7)	281	51.5	(47.2-55.7)
≥30	208	40.0	(35.8-44.4)	123	22.5	(19.1-26.3)
**Marital status**	**N= 293** (missing: 227)			**N=353** (missing: 195)		
Not currently married or in union	53	18.1	(13.9-23.0)	246	69.7	(64.6-74.4)
Currently married or in union	240	81.9	(77.0-86.1)	107	30.3	(25.6-35.4)
**Education***	**N= 360***			**N= 360** (missing = 2) *		
No formal education	222	61,7	(56.4-66.7)	3	0.8	(0.2-2.4)
Any primary education	100	27,8	(23.2-32.7)	98	27.2	(22.7-32.1)
Any secondary education	31	8,6	(5.9-12.0)	240	66.7	(61.5-71.5)
Any tertiary education	7	1,9	(0.8-4.0)	19	5.3	(3.2-8.2)
**Socio-economic status[a]***	**N=339** (missing: 21) *			**N=357** (missing: 5) *		
Low	115	33.9	(28.9-39.2)	156	43.7	(38.5-49.0)
Middle	192	56.6	(51.2-62.0)	184	51.5	(46.2-56.8)
High	32	9.4	(6.5-13.1)	17	4.8	(2.8-7.5)
**<u>Reproductive history and obstetric characteristics</u>**						
**Previous pregnancies**	**N=517** (missing: 3)			**N=547** (missing: 1)		
0	88	17.0	(13.9-20.5)	136	24.9	(21.3-28.7)
1 or more	429	83.0	(79.5-86.1)	411	75.1	(71.3-78.7)
**Gestational age (in weeks)** [b]	**N=488** (missing: 32)			**N=514** (missing: 34)		
Median (range)	*16 (4-28)*			*10 (4-28)*		
<13	188	38.5	(34.2-43.0)	345	67.1	(62.9-71.2)
13-28	300	61.5	(57.0-65.8)	169	32.9	(28.8-37.1)
**Severe complications (PLTC[c] + Near-miss[d] + Deaths)**	**N=520**			**N=548**		
Yes	348	66.9	(62.7-71.0)	278	50.7	(46.5-55.0)
**Type of complications (not mutually exclusive)**	**N=520**			**N=548**		
Severe bleeding/hemorrhage	374	71.9	(67.8-75.7)	317	57.8	(53.6-62.0)
Infection	97	18.7	(15.4-22.3)	148	27.0	(23.3-30.9)
Perforation	0	0.0	-	9	1.6	(0.8-3.1)
	**N=517 (missing: 3)**			**N=513 (missing: 35)**		
Anemia	424	82.0	(78.4-85.2)	219	42.7	(38.4-47.1)

* Only on the sub-sample of women who participated to the quantitative survey (N=362 in CAR and N=360 in Nigeria)

[a] Same SES composite indicator as in the WHO MCS-A study[1]. It was created from four questions from the quantitative survey. The four questions ask the woman: whether she has running water in her home; whether her household income allowed her to take care of all of her personal needs such as food and health during the past month; whether members of her household have been able to save money during the past month, after taking care of all household expenses; and whether she has had to go for a full day without a meal during the past month because of lack of food and inability to buy food in her household. For the first 3 questions, the answer “yes“ was coded “1“ and the answer “no“ was coded “0“. For the last question (full day without a meal), the answer “yes“ was coded “0“ and the answer “no“ was coded “1“. A score was created for each woman adding up the codes attributed to each of the 4 questions. A total of four indicates the highest SES and 0 the lowest.

Those with scores of 0 or 1 were categorized as low SES, those with 2 or 3 were categorized as middle SES, and those with scores of 4 were categorized as high SES.

[b] Gestational age was estimated in weeks of gestation using the ultra-sound assessment as the priority assessment method. For those missing this information, the following methods were used in order: the last menstrual period date, the uterine size assessed by the provider, or the provider's estimation of gestational age without mentioning the method was used.

[c] PLTC: Potentially life- threatening complications including severe hemorrhage, severe systemic infection or suspected uterine perforation according to WHO-MCS-A criteria[1]

[d] Near- miss case: organ dysfunction of either one or more of the following: cardiovascular, respiratory, renal, coagulation, hepatic, neurological or uterine dysfunction according to WHO (and WHO-MCS-A) criteria[1, 2]

References:

1. Qureshi Z, Mehrtash H, Kouanda S, Griffin S, Filippi V, Govule P, et al. Understanding abortion-related complications in health facilities: results from WHO multicountry survey on abortion (MCS-A) across 11 sub-Saharan African countries. BMJ Glob Heal. 2021;6:e003702.

2. World Health Organization, Organization WH. Evaluating the quality of care for severe pregnancy complications: the WHO near-miss approach for maternal health. Geneva: World Health Organization; 2011.
