# Supplementary material for: Assessing post-abortion care using the WHO quality of care framework for maternal and newborn health: a cross-sectional study in two African hospitals in humanitarian settings
Source: Reprod Health. 2024 Aug 5;21:114. doi: 10.1186/s12978-024-01835-9 (PMC11299292; doi:10.1186/s12978-024-01835-9)
Supplement: Supplementary file 5 — Additional file 5. Process (provision & experience of care) & outcome indicators measuring the quality of post-abortion care provided in the Nigerian and CAR hospitals. [file 12978_2024_1835_MOESM5_ESM.pdf]

**Additional file 5**: Process (provision & experience of care) & outcome indicators measuring the quality of post-abortion care provided in the Nigerian and CAR hospitals.

**Process - Provision of care**

| Indicators | Nigerian hospital | | | CAR hospital | | |
|---|---|---|---|---|---|---|
| **Coverage of key practices** | **n/N** | **Percentage %** | **95% CI** | **n/N** | **Percentage %** | **95% CI** |
| **Uterine evacuation management** | | | | | | |
| Any uterine evacuation | 485/520 | 93.3% | 90.8-95.3 | 487/548 | 88.9% | 85.9-91.4 |
| Uterine evacuation by uterotonic | 474/520 | 91.2% | 88.4-93.5 | 267/548 | 48.7% | 44.5-53.0 |
| Instrumental uterine evacuation (i.e., uterine evacuation using any types of instruments: MVA, EVA, D&E, D&C) | 93/520 | 17.9% | 14.7-21.5 | 387/548 | 70.6% | 66.6-74.4 |
| **Other medical management of severe complications** | | | | | | |
| Blood transfusion | 219/520 | 42.1% | 37.8-46.5 | 30/548 | 5.5% | 3.7-7.7 |
| IV fluids | 370/520 | 71.2% | 67.1-75.0 | 281/548 | 51.3% | 47.0-55.5 |
| Admission in critical care unit (Intensive Care Unit in Nigeria and High Dependency Unit in CAR[&]) | 31/520 | 6.0% | 4.1-8.4 | 31/548 | 5.7% | 3.9-7.9 |
| Hysterectomy | 1/520 | 0.2% | 0.0-1.1 | 5/548 | 0.9% | 0.3-2.1 |
| **Pain management** | | | | | | |
| Painkillers in all patients | 188/520 | 36.2% | 32.0-40.5 | 434/548 | 79.2% | 75.6 - 82.5 |
| **Contraception uptake at discharge** | | | | | | |
| Contraception uptake in medically discharged | 23/495 | 4.7% | 3.0-6.9 | 301/ 475[§] | 63.4% | 58.9 - 67.7 |
| **Actionable information system: completeness of clinical medical record** | **n/N** | **Percentage %** | **95% CI** | **n/N** | **Percentage %** | **95% CI** |
| Women with complete key pieces of information in their medical record[Ω] | 390/520 | 75.0% | 71.1-78.7 | 332/548 | 60.6% | 56.4-64.7 |

**Process – Provision of care (to be continued)**

| Indicators | Nigerian hospital | | | CAR hospital | | |
|---|---|---|---|---|---|---|
| **Application of post-abortion care evidence-based clinical guidelines** | **n/N** | **Percentage %** | **95% CI** | **n/N** | **Percentage %** | **95% CI** |
| **Uterine evacuation management** | | | | | | |
| Dilatation & (sharp) Curettage (D&C) among women who had an instrumental uterine evacuation | 1/93 | 1.1% | 0.0 - 5.8 | 9/387 | 2.3% | 1.1 - 4.4 |
| **Blood transfusion** | | | | | | |
| Blood transfusion when indicated[π] | 95/97 | 97.9% | 92.7-99.7 | 25/30 | 83.3% | 65.3 - 94.4 |
| Blood transfusion with no documented indication[π] | 124/423 | 29.3% | 25.1-33.9 | 5/518 | 1.0% | 0.3 - 2.2 |
| **Infection prevention and management** | | | | | | |
| (Curative) Antibiotics among septic abortions[Σ] | 68/80 | 85.0% | 75.3-92.0 | 117/145 | 80.7% | 73.3– 86.8 |
| (Prophylactic) Antibiotic when having an instrumental or surgical intervention | 92/96 | 95.8% | 89.7-98.9 | 157/392 | 40.1% | 35.2-45.1 |
| Antibiotic with no documented indication[Ψ] | 99/358 | 27.7% | 23.1-32.60 | 35/115 | 30.4% | 22.2– 39.7 |
| Tetanus immunization status checked and managed adequately among all women | 10/520 | 1.9% | 0.9-3.5 | 38/548 | 6.9% | 5.0 – 9.4 |
| **Pain management** | | | | | | |
| Anesthesia (para cervical block, spinal anesthesia, general anesthesia) in instrumental uterine evacuation | 83/93 | 89.3% | 81.1-94.7 | 238/387 | 61.5% | 56.4 - 66.4 |
| **Iron/Folic acid at discharge** | | | | | | |
| Iron in patients with anemia medically discharged | 378/403 | 93.8% | 91.0-96.0 | 206/208 | 99.0% | 96.6-99.9 |
| Folic acid in patients with anemia medically discharged | 373/403 | 92.6% | 89.5-94.9 | 206/208 | 99.0% | 96.6-99.9 |
| **Contraception counseling at discharge** | | | | | | |
| Contraception counselling in medically discharged | 195/495 | 39.4% | 35.1-43.9 | 509/516[μ] | 98.6% | 97.2 - 99.5 |
| **Composite indicator of application of post-abortion care evidence-based practices** | | | | | | |
| **Poor key evidence-based practices[Δ]** | **213/520** | **41.0%** | **36.7-45.3** | **292/548** | **53.3%** | **49.0-57.5** |

| | Indicators | Nigerian hospital | | | CAR hospital | | |
|---|---|---|---|---|---|---|---|
| Process - Experience of care | **Reported Experience of post-abortion care** | **n** | **Percentage %** | **95% CI** | **n** | **Percentage %** | **95% CI** |
| | **Effective communication** | N=359[µ] | | | N= 361[µ] | | |
| | During your stay at this hospital, were you given explanations regarding your care and treatment? | 177 | 49.3 | 44.0-54.6 | 214 | 59.3 | 54.0-64.4 |
| | Were you able to ask questions during examination and treatment? | 59 | 16.4 | 12.8-20.7 | 53 | 14.7 | 11.2-18.8 |
| | **Respect and preservation of dignity** | N=359[µ] | | | N= 361[µ] | | |
| | Were you spoken to nicely? | 315 | 87.7 | 83.9-91.0 | 286 | 79.2 | 74.7-83.3 |
| | Did you receive pain medications during your hospital stay? | 287 | 79.9 | 75.4-84.0 | 296 | 82.0 | 77.6-85.8 |
| | During your physical examination, were you covered up (or given privacy in such a way that only the doctor or nurse examining you could see you)? | N=358[α] | | | N= 361[µ] | | |
| | Yes, all the time | 309 | 86.3 | 82.3-89.7 | 134 | 37.1 | 32.1-42.3 |
| | Yes, most of the time | 21 | 5.9 | 3.7-8.8 | 154 | 42.7 | 37.5-47.9 |
| | Yes, a few times | 16 | 4.5 | 2.6-7.2 | 48 | 13.3 | 10.0-17.2 |
| | Never | 6 | 1.7 | 0.6-3.6 | 21 | 5.8 | 3.6-8.7 |
| | Not examined | 6 | 1.7 | 0.6-3.6 | 4 | 1.1 | 0.3-2.8 |
| | How do you feel about the amount of time you waited to see a health provider? | N=358[α] | | | N= 358[β] | | |
| | Very short | 251 | 70.1 | 65.1-74.8 | 96 | 26.8 | 22.3-31.7 |
| | Somewhat short | 41 | 11.5 | 8.3-15.2 | 125 | 34.9 | 30.0-40.1 |
| | Somewhat long | 41 | 11.5 | 8.3-15.2 | 64 | 17.9 | 14.0-22.2 |
| | Very long | 25 | 7.0 | 4.6-10.1 | 73 | 20.4 | 16.3-24.9 |
| | **Composite indicator of reported experience of post-abortion care** | N=358[α] | | | N= 358[β] | | |
| | **Number of poor experiences of care** | | | | | | |
| | <2 poor experiences | **113** | **31.6** | **26.8-36.7** | **63** | **17.6** | **13.8-21.9** |
| | >= 2 poor experiences | **245** | **68.4** | **63.3-73.2** | **295** | **82.4** | **78.1-86.2** |

| | Indicators | Nigerian hospital | | | CAR hospital | | |
|---|---|---|---|---|---|---|---|
| | | n | Percentage % | 95% CI | n | Percentage % | 95% CI |
| **Outcomes** | **People-centered outcome** | N=359^µ | | | N= 361^µ | | |
| | Did you feel the doctors, nurses and other staff at the facility took the best care of you? | | | | | | |
| | Yes, all the time (optimal care) | 232 | 64.6 | 59.4-69.6 | 124 | 34.3 | 29.5-39.5 |
| | No, not all the time (no optimal care) | 127 | 35.4 | 30.4-40.6 | 237 | 65.7 | 60.5-70.5 |
| | **Abortion-related health outcomes (definitions in Supl. table 1)** | **n/N** | **Percentage %** | **95% CI** | **n/N** | **Percentage %** | **95% CI** |
| | **Abortion-related mortality index** (Original WHO near-miss criteria†) | 1/24 | 4.2 | 0.1-21.1 | 2/21 | 9.5 | 1.2-30.4 |
| | **Abortion-related mortality index** (WHO near-miss criteria adapted for Sub-Saharan Africa†) | 1/103 | 1.0 | 0.0-5.3 | 2/34 | 5.9 | 0.7-19.7 |
| | **Risk of healthcare-related near-miss** (Original WHO near-miss criteria†) | 1/520 | 0.2 | 0.0-1.1 | 6/548 | 1.1 | 0.4-2.4 |
| | **Risk of healthcare-related near-miss** (WHO near-miss criteria adapted for Sub-Saharan Africa†) | 7/520 | 1.3 | 0.5-2.6 | 12/548 | 2.1 | 1.1-3.8 |

^Ω All the following key pieces of information are available in their medical record: estimate of gestational age, information on vital signs (temperature, systolic & diastolic blood pressures, heart rate, respiratory rate), abdominal examination, cervix examination, mental status, appearance at presentation & final diagnosis.

^& An intensive care unit (ICU) is a unit that provides 24-hour medical supervision (including continuous vital signs monitoring), mechanical ventilation (including oxygen) and continuous vaso-active drugs. The High Dependency Unit (HDI) is a unit with all characteristics except the mechanical ventilation.

^π A woman who had an indication of blood transfusion is defined by (MSF guidelines 2019(1)) a woman with:

- Hb ≤ 5 g/dl, even if there are no signs of decompensation
- Hb > 5 g/dl and < 7 g/dl if there are signs of decompensation (lowest SBP <= 90 mm Hg & pulse >= 100 b/min) or sickle cell disease or severe malaria or serious bacterial infection or pre-existing heart disease.

^Σ Septic abortions include uterine infection, generalized peritonitis or severe systemic infection with genital origin and exclude extra-genital infections (malaria, urinary infections, etc.)

^Ψ No documented indication of antibiotics includes: no documented 1- infection, 2- instrumental/surgical procedure, 3- trauma/perforation (no evidence of cervix/vaginal mechanical injury at clinical examination, uterine perforation or other intra-abdominal perforation confirmed at laparotomy or at clinical examination), 4- notion of septic maneuver to induce abortion and 5- foreign body found in the vagina.

^Δ Get at least one of these interventions not following MSF guidelines: get D&C or did not get antibiotics in septic abortions or did not get antibiotic in instrumental or surgical treatment or get antibiotic when not indicated or did not get blood transfusion when indicated or get blood transfusion when not indicated.

§ Missing data: n=42; µ Missing data: n=1; α Missing data: n=2: β Missing data: n=4

† Near- miss cases: women with organ dysfunction of either one or more of the following: cardiovascular, respiratory, renal, coagulation, hepatic, neurological or uterine dysfunction using 1) original WHO near-miss criteria(2) to allow comparison with literature & 2) WHO near-miss criteria adapted for Sub-Saharan Africa(3) as a sensitivity analysis.

MVA: Manual Vacuum Aspiration, EVA: Electric Vacuum Aspiration, D&E: Dilatation & Evacuation, D&C: Dilatation & (sharp) curettage (not recommended)

**References:**


1. MSF. Essential Obstetric and Newborn care : Practical guide for midwives, doctors with obstetrics training and health care personnel who deal with obstetric emergencies [Internet]. Medecins Sans Frontieres. 2019. 252 p. Available from: https://medicalguidelines.msf.org/en/viewport/ONC/english/essential-obstetric-and-newborn-care-51415817.html
2. World Health Organization, Organization WH. Evaluating the quality of care for severe pregnancy complications: the WHO near-miss approach for maternal health [Internet]. WHO. Geneva: World Health Organization; 2011 [cited 2018 Apr 11]. Available from: www.who.int/reproductivehealth
3. Estelle Pasquier, Onikepe O. Owolabi, Tamara Fetters, Richard Norbert Ngbale, Mariette Claudia Adame Gbanzi, Timothy Williams, et al. High severity of abortion complications in fragile and conflict affected settings: A cross-sectional study in two referral hospitals in Sub-Saharan Africa (AMoCo study). BMC Pregnancy Childbirth [Internet]. 2023;6(143):1–15. Available from: https://doi.org/10.1186/s12884-023-05427-6
